# On the quest for novel bio-degradable plastics for agricultural field mulching

**DOI:** 10.3389/fbioe.2022.922974

**Published:** 2022-08-08

**Authors:** Sami Ullah Dar, Zizhao Wu, Linyi Zhang, Peirong Yu, Yiheng Qin, Yezi Shen, Yunfan Zou, Leslie Poh, Yoav Eichen, Yigal Achmon

**Affiliations:** ^1^ Biotechnology and Food Engineering, Guangdong Technion—Israel Institute of Technology, Shantou, China; ^2^ Guangdong Technion Department of Chemistry—Israel Institute of Technology, Shantou, China; ^3^ Polymer Physics Laboratory, Department of Chemical Engineering, Guangdong Technion–Israel Institute of Technology (GTIIT), Shantou, China; ^4^ Faculty of Biotechnology and Food Engineering, Technion—Israel Institute of Technology, Haifa, Israel; ^5^ Guangdong Provincial Key Laboratory of Materials and Technologies for Energy Conversion, Guangdong Technion—Israel Institute of Technology, Shantou, China

**Keywords:** plastic biodegradation, soil respiration, microbial volatile organic compounds, PTR-ToF-MS (proton transfer reaction time-of-flight mass spectrometry), disulfide-based polymer binder

## Abstract

Plasticulture, the practice of using plastic materials in agricultural applications, consumes about 6.7 million tons of plastics every year, which is about 2% of the overall global annual plastics production. For different reasons, plastic material used for agriculture is difficult to recycle. Therefore, most of it is either buried in fertile soils, thereby significantly causing deterioration of their properties, or, at best case, end in landfills where its half-life is measured in decades and even centuries. Hence, developing biodegradable plastic materials that are suitable for agricultural applications is a vital and inevitable need for the global human society. In our labs, two types of potentially biodegradable plastic polymer films were prepared and characterized imidazolium in terms of their bio-degradability. In the first approach, polymers made of ionic liquid monomers were prepared using photo radical induced polymerization. The second approach relies on formation of polyethylene-like n-alkane disulfide polymers from 1,ω-di-thiols through thermally activated air oxidation. These two families of materials were tested for their biodegradability in soils by using a simulation system that combines a controlled environment chamber equipped with a respirometer and a proton-transfer-reaction time of flight mass spectrometer (PTR-TOF-MS) system. This system provides a time-dependent and comprehensive fingerprint of volatiles emitted in the degradation process. The results obtained thus far indicate that whereas the ionic-liquid based polymer does not show significant bio-degradability under the test conditions, the building block monomer, 1,10-n-decane dithiol, as well as its disulfide-based polymer, are bio-degradable. The latter reaching, under basic soil conditions and in room temperature, ∼20% degradation within three months. These results suggest that by introduction of disulfide groups into the polyethylene backbone one may be able to render it biodegradable, thus considerably shortening its half-life in soils. Principal component analysis, PCA, of the data about the total volatiles produced during the degradation in soil indicates a distinctive volatile “fingerprint” of the disulfide-based bio-degradable products which comes from the volatile organic compounds portfolio as recorded by the PTR-TOF-MS. The biodegradation volatile fingerprint of this kind of film was different from the “fingerprint” of the soil background which served as a control. These results can help us to better understand and design biodegradable films for agricultural mulching practices.

## Introduction

Plastic waste is an increasing concern around the world. The use of plastics in agricultural practices such as mulching is on the rise as well (Huang et al., 2020). Plasticulture, i.e. agricultural practices that include the use of plastic materials, is essential for food security in most parts of the world. The usage of plastic polymers in agriculture is quite diverse, including for example: greenhouse covers, trailing, packaging, irrigation systems, silage, soil mulching and more (Briassoulis and Dejean, 2010; Zumstein et al., 2019; Zurier and Goddard, 2021). Although necessary, the use of plastics in agriculture can lead to wide environmental adverse effects, such as contamination of ground water, disturbance of the ecosystems of terrestrial and aquatic fauna and flora (Zhou et al., 2020), deterioration of soil properties of agricultural lands (for example their gas exchange and water retention capacity), spread of toxic microplastics particles (Sander, 2019; Huang et al., 2020; Zurier and Goddard, 2021) etc. Plastic mulching is probably the most abundant plasticulture practice. Agricultural plastic mulching is done for various reasons, including water preservation, crop protection, soil remediation, treatments against weeds and soilborne pathogens (such as solarization and fumigation (Achmon et al., 2017)) and other important practices used to increase the yields of crops. Mulching is estimated to cover an agricultural area of more than 128,500 km^2^ around the world (Zhou et al., 2020) and China is the global leader in terms of plasticulture mulching practices (Huang et al., 2020). For these reasons, the need for biodegradable plastics for agricultural mulching is critical from the environmental point of view. Although it is an obvious need, not many commercial solutions are currently available and most of the agricultural mulching done today utilizes non-degradable plastic polymers, mainly polyethylene (PE), (Briassoulis et al., 2015; Ahmed et al., 2018; Zhang et al., 2019). Unlike the slow implementation of biodegradable plastic polymers in commercial agricultural mulching systems, research in this field receives increased attention and has a fast progress pace (Kyrikou and Briassoulis, 2007; Sander, 2019; Huang et al., 2020). There are several important characteristics that plastic mulching materials must possess along their entire service life in order to be widely applied in the field: 1) durability (mechanical, chemical, resistance to water and solvents, etc.), 2) mechanical flexibility (ability to be stretched over various shapes of field plots), 3) light weight, 4) modularity (can have variable thickness, color and gas permeability), 5) non-toxicity, and most important, 6) affordability (being inexpensive and easy to produce in very large quantities). The combination of these characteristics makes it hard to find alternative biodegradable plastic polymers to replace the current non-degradable ones. Moreover, it is challenging to define what can be considered as a biodegradable plastic in soil and what is only bio-available (a material that judging from its chemical formula looks biodegradable, but does not actually degrade in a relevant pace (Zumstein et al., 2019)). Some recent progress was done in this area by introducing the European Standard EN 17033: Plastics–Biodegradable mulch films (EN17033 Plastics, 2018). Additionally, most degradation processes are complex and are not sufficiently characterized, and hence are less understood from microbiological and chemical perspectives (Sander, 2019). Yet there are some examples of biodegradable mulching materials that are available, such as cellulose, starch, poly-b-hydroxybutyrate and alike (Kyrikou and Briassoulis, 2007; Sander, 2019). To date, most polymer soil biodegradability studies have been using standard methods such as respiration or polymer size reduction (Achmon et al., 2019) to assess the biodegradability rate. Some recent studies used ^13^C-labeled polymers to closely monitor the degradation of the carbon skeleton of the polymer (Thomas et al., 2021). The analysis of biodegradability is still a debatable issue and although standards are available and are being updated (EN17033 Plastics, 2018), there is still a lot of room for improvement. Generally, the biodegradability can be looked upon through the carbon conversion by Equation 1.

Equation 1:

$$C_{n}^{\mathrm{polymer}}\mathrm{+x}O_{2}\to C_{\mathrm{n-}\left(\mathrm{x+y}\right)}^{\mathrm{biomass}}\mathrm{+xC}O_{2}+C_{y}^{\mathrm{residues}}$$ (1)

Where C^polymer^ is the polymers’ carbon backbone, C^biomass^ is the utilization of the carbon to build live biomass (mainly microbial) and C^residues^ represents the carbon remaining in polymer residues as long as biodegradation is not completed (Sander, 2019). However, a more precise equation is presented in Equation 2.

Equation 2:

$$C_{n}^{\mathrm{polymer}}\mathrm{+x}O_{2}\to C_{\mathrm{n-}\left(\mathrm{x+y+z}\right)}^{\mathrm{biomass}}\mathrm{+xC}O_{2}+C_{y}^{\mathrm{residues}}+C_{z}^{\mathrm{secondary products}}$$ (2)

Where C^secondary products^ are additional secondary metabolites produced by those microbes that are not converting all of the polymer into biomass and emit secondary products back to the environment. In most of the studies on biodegradability of polymers the focus is on the CO_2_ and C^polymer^, and sometimes on C^residues^, but rarely on C^secondary products^. C^secondary products^ can also be divided according to Equation 3.

Equation 3:

$$C_{z}^{\mathrm{secondary}\;\mathrm{products}}=C_{a}^{\mathrm{non-volatile}\;\mathrm{products}}+C_{b}^{\mathrm{volatile}\;\mathrm{products}}$$ (3)

Only few studies have looked at the C^non-volatile products^ (Zumstein et al., 2019; Thomas et al., 2021) which are mainly soluble residues secreted by the microbial activity, and as far as the authors know, no study to date has looked at the C^voltile products^ emanating by the polymer’s biodegradation process in the soil.

In this study we tested two new synthetic materials as potential candidates to be used as biodegradable plastic polymers for agricultural mulching. We also report here a first insight into C^voltile products^ (Volatile Organic Compounds (VOCs)) emitted during the biodegradation process of those two new types of polymers. This study is opening a hatch to a new way of looking at the complex mechanisms underlying the biodegradation of polymers in the soil through VOCs production during the process.

## Materials and methods

### Apparatus

Respirometery experiments were performed using a Micro-Oxymax Respirometer (Columbus Instruments, United States). Detectors of CO_2_ Carbon Dioxide Sensor 0–10% CH_4_ Methane Measuring (0–10%) and O_2_ Paramagnetic Oxygen Sensor 0–100% - (Columbus Instruments, United States) where used to monitor the simulated process of the biodegradation of plastics in soil. Mass spectrometry was performed using a PTR-TOF-MS 1000 (Ionicon Analytik Ges.m.b.H., Innsbruck, Austria).

## Materials

Ethyl bromoacetate, 1-vinyl imidazole, butylated hydroxyl toluene (BHT), 1,10-decanedithiol, sodium hydroxide (NaOH), phenylbis (2,4,6-triethylbenzoyl) phosphine oxide were purchased from Sigma-Aldrich (Merck) and used as received. Microcrystalline cellulose was purchased from Alfa Aesar Co. and used as received. All solvents, such as ethyl acetate, methanol, THF and acetone, as well as other materials used in the present research were purchased from Shanghai Macklin Biochemical Co., and were of the highest purity available. All commercial materials were used as received unless specifically noted. Tryptic Soy Agar (TSA) was purchased from BD, MD, United States. Oxytetracycline-Glucose Yeast Extract (OGYE) Agar Base was purchased from Solarbio, Beijing, China. Oxytetracycline hydrochloride was purchased from Solarbio, Beijing China. *Bacillus cereus* (BC) Agar Base was purchased from OXOID, Basingstoke, United Kingdom. Eosin Methylene Blue Agar (EMBA) was purchased from HuanKai Microbial, Guangdong, China.

### 1-vinyl-3-(2-ethoxy-2-oxoethyl) imidazolium bromide

Ethyl bromoacetate (10.0 ml, 225 mmol) and 50.0 ml of ethyl acetate were added to a 100 ml round bottom flask equipped with a magnetic stirrer. 1-vinyl imidazole (8.17 ml, 90.2 mmol) containing 100 ppm butylated hydroxyl toluene (BHT) was added dropwise to the flask while stirring, and the mixture was left to stir for 24 h at room temperature. The precipitate was filtered and washed with three portions of ethyl acetate, then dried in a vacuum oven at 40°C for 24 h. The product, 1-vinyl-3-(2-ethoxy-2-oxoethyl) imidazolium bromide, in the form of an off-white solid, was obtained with an 81% yield.

Off white solid, M.P. = 95°C; 81% yield; ^1^H NMR (400 MHz, D_2_O, δ): 9.10 (s, 1H, 2-Im), 7.80 (s, 1H, 4-Im), 7.57 (s, 1H, 5-Im)), 7.11 (dd, J = 16.0, 8.0 Hz, 1H, N-C**H** = CH_2_), 5.78 (dd, J = 16.0, 4.0 Hz, 1H, CH = C**H**_2_), 5.42 (dd, J = 8.0, 8.0 Hz, 1H, CH = C**H**_2_), 5.21 (s, 2H, -N^+^C-C**H**_2_-C=O-), 4.23 (q, J = 6.0 Hz, 2H, CH_3_-C**H**_2_-O), 1.20 (t, J = 8.0 Hz, 3H, -CH_2_-C**H**_3_); ^13^C NMR (400 MHz, D_2_O, δ): 167.86, 135.95, 128.18, 124.23, 119.47, 110.30, 63.74, 50.35, 13.32; HRMS (MALDI-TOF) (ESI) *m/*z: [M-Br]^+^calc. for C_9_H_13_N_2_O_2_, 181.216; found, 181.052.

### Poly-(1-vinyl-3-(2-ethoxy-2-oxoethyl) imidazolium bromide)

15 uL of photoinitiator stock solution (0.3 g of phenylbis (2,4,6-triethylbenzoyl) phosphine oxide in 1 g of THF) was added to 1 g of a 1:1 w/v liquid mixture of 1-vinyl-3-(2-ethoxy-2-oxoethyl) imidazolium bromide and 1-vinyl imidazole. The combined solution was spread over glass slides and exposed to UV-VIS light to induce polymerization. The product is obtained in the form of a yellowish-brown film (Sevilia et al., 2022; Zertal et al., 2022).

### Poly 1,10-disulfanyl-n-decane

1,10-decanedithiol (9.5 ml, 43.7 mmol) was added to a solution of NaOH (18 g, 450 mmol) in methanol (300 ml) using a 500 ml round bottom flask equipped with a compressed air inlet and an outlet. The reaction mixture was stirred vigorously for 5 days with the addition of 250 ml of methanol each day to compensate for evaporation. The crude, in the form of white color suspension with some lumps, was dried and washed with several portions of water until it became neutral, then with several portions of methanol and acetone, and then dried in a vacuum oven at 45 °C for 48 h. The product, in the form of a white solid, was obtained with a 54% yield (Tada et al., 2012).

White solid, 54% yield; ^1^H NMR (400 MHz, CDCl_3_, δ): 2.66–2.49 (t, q, J = 4 Hz, J = 8 Hz 8H, -**H**_2_C-S-S-C**H**_2_-), 1.60–1.67 (complex multiplet, 8H, -C**H**_2_-CH_2_-S-S-CH_2_-C**H**_2_-), 1.28–1.39 (complex multiplet, 24H, central alkyl chain); ^13^C NMR (400 MHz, CDCl_3_, δ): 39.17, 34.03, 29.4–29.43, 28.36–28.52, 24.65.

### Determination of inherent biodegradability

The biodegradability of polymer poly-(disulfanyl-n-decane)1,10 decanedithiol (carbon content: 58.8%wt.) and of monomer 1,10-decane dithiol (carbon content: 58.2%wt.) in soil under basic conditions and unadjusted conditions was investigated in this preliminary experiment. The biodegradability of plastic materials was determined according to **ISO 17556:2019** (ISO, 2019) by measuring the amounts of evolved CO_2_. Soil samples were collected from the field experiment station of the GTIIT, in Shantou, Guangdong, China (23°31′4″N, 116°45′6″E). After collection, the soil was sieved to give particles smaller than 2 mm in size and obvious plant materials, stones and other inert materials were removed. The sieved soil was then air-dried under the sun for three days. In the test, a biodegradable reference material, microcrystalline cellulose (Alfa Aesar Co. Inc.), was used as a positive control. Samples were prepared by thoroughly mixing 60 g of dry soil with 0.6 g polymer/monomer/reference material. 24 g of distilled water were added to each sample to reach approximately 50% total water-holding capacity. To adjust the sample to the basic environment, 4.2 ml NaOH solution (1 M) was added to the samples at the beginning of the experiment. Throughout the experiment, samples’ pH was maintained at 8.5–8.8 for basic groups and at 5.0–5.5 for unadjusted groups. Soil with only water addition served as the blank group of the test. The soil mixtures were placed inside 100 ml flasks which were connected to a Micro-Oxymax Respirometer (Columbus Instruments, United States). The system was set in aerobic mode and was capable of measuring CO_2_, O_2_ and CH_4_ quantities (CO_2_ Sensor 0–10% CH_4_ Measuring (0–10%) and O_2_ Paramagnetic Sensor 0–100% - Columbus Instruments, United States) with a cycle of approximately 4 h for each individual flask. All flasks were incubated in triplicates at 25°C. Every 10 days, water was added to keep the water content between 40 and 60%wt. of the total water-holding capacity and the soil was gently re-mixed.

The amount of CO_2_ evolved was measured by the Micro-Oxymax Respirometer and the data was collected with the help of the Micro-Oxymax Windows software. Since the amount of CO_2_ was recorded in volume units, the measured mass of CO_2_ (m) was calculate by using the ideal gas law, Equation 4, and Equation *5*.

Equation 4:

$$\mathrm{PV=nRT=}\frac{m}{\mathrm{Mw}}\mathrm{RT}$$ (4)

Equation 5:

$$\mathrm{m=}\frac{\mathrm{PVMw}}{\mathrm{RT}}$$ (5)

where V is the volume, *P* is the pressure in the respirometer (811 mmHg), R is the ideal gas constant, T is the system temperature (298°K), V is the measured volume of CO_2_, and Mw is the molecular weight of CO_2_ (44.01 g/mol).

In accordance with **ISO 17556:2019**, the percentage biodegradation (D_t_) was calculated from Equation 6 and Equation *7*:

Equation 6:

$$\mathrm{ThC}O_{2}=\frac{\mathrm{44}}{\mathrm{12}}\times M_{T}\times w_{C}$$ (6)

Equation 7:

$$D_{t}=\frac{\sum m_{T}-\sum m_{B}}{\mathrm{ThC}O_{2}}\times 100$$ (7)

where ThCO_2_ is the theoretical amount of CO_2_ that can be evolved by the test material, M_T_ is the mass of test material introduced into each flask, w_c_ is the carbon content of the test material determined from the chemical formula, Σm_T_ is the cumulative amount of CO_2_ evolved in the flask containing test material and soil mixture, Σm_B_ is the cumulative amount of CO_2_ evolved in the flask containing mere soil (blank). The test was done until 60% of the reference material, microcrystalline cellulose, was degraded given what can be named “inherent biodegradability” that indicates the potential of the materials to be bioavailable in a relatively short period of time.

### PTR-TOF-MS analysis

Samples were prepared in 100 ml flasks as done for the biodegradability test at 25°C. An additional abiotic group was prepared by placing 0.9 g of plastics inside an empty flask. The flasks were capped with screw caps having two channels, one for aeration and one for injection into the apparatus. The headspace composition of the samples was measured using a PTR-TOF 1000 (Ionicon Analytik Ges.m.b.H., Innsbruck, Austria) under the following working conditions: 630 V drift voltage, 2.30 mbar drift pressure, 6.5 sccm inlet flow, 80°C drift tube and inlet temperature. H_3_O^+^ was employed as the primary ion and the instrument was operated at an E/N ratio (E corresponds to the electric field strength and N to buffer gas number density within the drift tube) of 142 Td (1 Td = 10^–17^ Vcm^2^). For each flask, more than 120 cycles (2 min each) were recorded, and a stable stage (generally 90 to 120 cycles) was used for the analysis. All the samples were measured once a day.

### Microbial cultures of soil samples

A microbial test was done for microbial load in the tested samples. Four kinds of culture media were prepared for testing the microbial culture growth: Tryptic Soy Agar (TSA) (BD, MD, United States), Oxytetracycline-Glucose Yeast Extract (OGYE) Agar Base (Solarbio, Beijing, China)) with the supplement of Oxytetracycline hydrochloride (Solarbio, Beijing China), *Bacillus cereus* (BC) Agar Base (OXOID, Basingstoke, United Kingdom) and Eosin Methylene Blue Agar (EMBA) (HuanKai Microbial, Guangdong, China). All media were prepared and sterilized according to their corresponding specifications. For each soil sample, 2 g of soil mixtures were transferred to 20 ml sterilized saline, followed by mixing using a vortex. After the dirt grains settled down, the supernatant (addressed as a 10^–1^ dilution) was collected and diluted to 10^–2^. For each medium, 0.1 ml dilution was used for plating. TSA and EMBA were incubated at 37°C for 24 h, while OGYE and BC were incubated at 30°C for 24 h.

### Data analysis

Statistical ANOVA tests were performed using the R 4.1.1 software (R Foundation for Statistical Computing, Vienna, Austria) to evaluate the differences in the CO_2_ evolution rate between plastic/reference material degradation processes and their corresponding blanks. The PTR data were pre-processed and analyzed using PTR-MS Viewer 3.2 (Ionicon Analytik Ges.m.b.H., Innsbruck, Austria). Principal component analysis (PCA) was carried out using R 4.1.1, and 3D plots of the PTR spectra were generated using MATLAB R2021b (The MathWorks, Inc., Natick, MA, United States), based on the exported PTR data.

## Results and discussion

### Polymers

**FIGURE 1:**
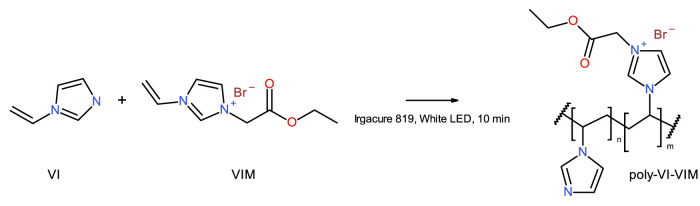
Synthesis of poly-VI-VIM copolymer, n/m = 3.75.

Two prototypes of polymer backbones were investigated, with the aim of developing novel biodegradable polymers, based on their biodegradability properties. A first prototype, poly-VI-VIM, is composed of a mixture of 1-vinylimidazole (VI) and 1-vinyl-3-(2-ethoxy-2-oxoethyl) imidazolium bromide (VIM), and was prepared by photoinduced radical polymerization, according to Figure 1, where n/m = 3.75. A 1:1 wt. mixture of VI and VIM was prepared, then 0.0045 mg/ml of photo initiator (Irgacure 819) was added, and the mixture was well mixed, spreaded atop a glass plate and cured using a 100 W white LED. Curing was monitored by FT-IR, and irradiation proceeded untill the complete disappearance of the vinyl absorption bands. The resulting films were used as prepared for the biodegradation experiments.

**FIGURE 2:**
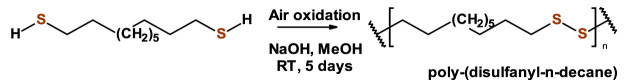
Air oxidation of 1,10-decanedithiol to poly-(disulfanyl-n-decane). Measuring the biodegradation of the polymers in the soil.

The second prototype material, poly-(disulfanyl-n-decane), was prepared in a single step from commercially available 1,10-decanedithiol through air oxidation under basic conditions, (Figure 2). 1,10-decanedithiol was dissolved in methanol containing NaOH and exposed to air flow for 5 days at room temperature. A white color thick melt was obtained, which was washed with several portions of distilled water until it became neutral, then washed with several portions of methanol and acetone, and then it was filtered and dried in a vacuum oven at 45°C for 48 h. The bright white powder, poly-(disulfanyl-n-decane), was used for the preparation of disk samples, Φ = 25 mm, thickness = 2 mm, through compression molding, done for 1 h at 125°C under 13 Mpa pressure between two smooth PTFE sheets.

**FIGURE 3:**
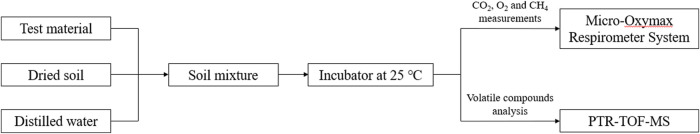
Flow chart of the biodegradability test and volatile compounds analysis. Test plastic (or reference material) and dried soil were mixed homogeneously with the supplement of distilled water. The soil mixture was incubated in a low temperature incubator with a constant temperature of 25 °C. The changes of CO_2_, O_2_ and CH_4_ concentrations were measured by a Micro-Oxymax Respirometer System in real time and the volatile compounds were analyzed using a PTR-TOF-MS.

Biodegradability of plastic polymers in the soil is usually measured by monitoring the emission of CO_2_ during the process (Briassoulis et al., 2020; Huang et al., 2020; Zurier and Goddard, 2021). In this study the Micro-Oxymax respirometer system was used to monitor the CO_2,_ O_2_ and CH_4_ evolution caused by the biodegradation of the two polymers poly-VI-VIM and poly-(disulfanyl-n-decane), in the soil (Figure 3).

**FIGURE 4:**
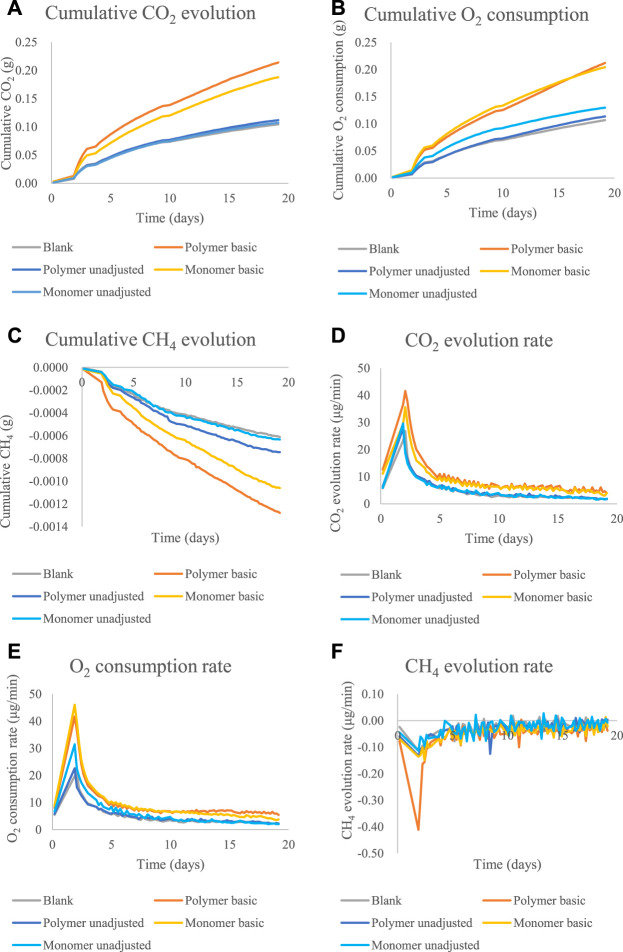
The cumulative amounts and rate of CO_2_, O_2_ and CH_4_ emission for the biodegradability test of poly-(disulfanyl-n-decane) and monomer 1,10-decane dithiol under basic conditions (pH∼8.5) and unadjusted conditions (pH∼5.3): **(A)** the cumulative CO_2_ evolution. **(B)** The cumulative O_2_ consumption. **(C)** The cumulative CH_4_ evolution. **(D)** The CO_2_ evolution rate. **(E)** The O_2_ consumption rate. **(F)** The CH_4_ evolution rate.

**FIGURE 5:**
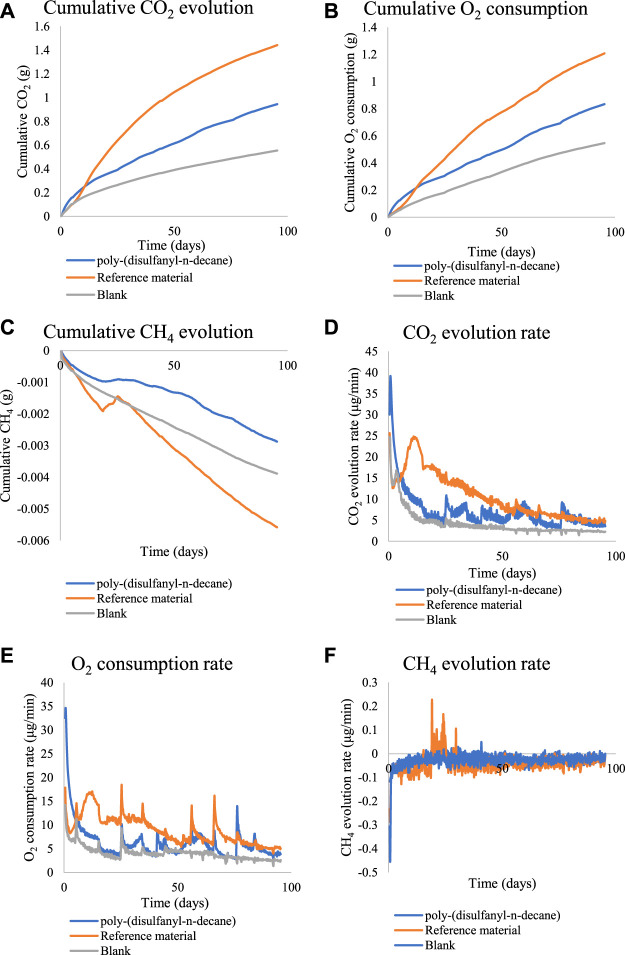
The cumulative amounts and rate of CO_2_, O_2_ and CH_4_ for the biodegradability test of poly-(disulfanyl-n-decane). **(A)** The cumulative CO_2_ evolution. **(B)** The cumulative O_2_ consumption. **(C)** The cumulative CH_4_ evolution. **(D)** The CO_2_ evolution rate. **(E)** The O_2_ consumption rate. **(F)** The CH_4_ evolution rate.

**FIGURE 6:**
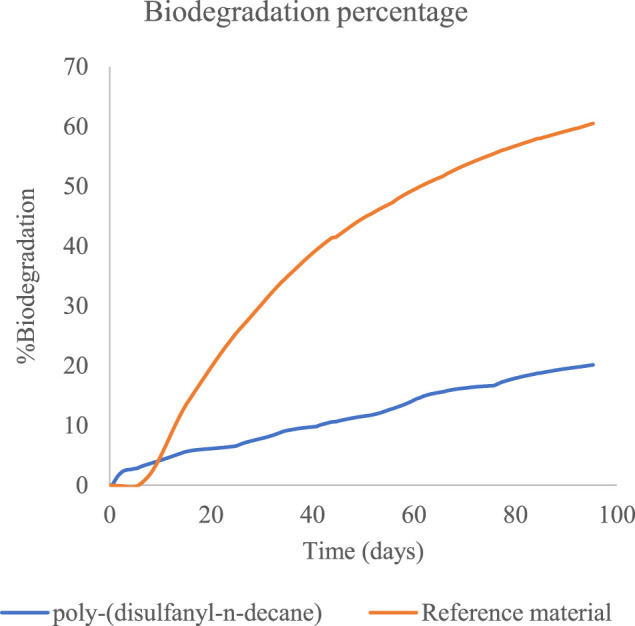
The percentage of biodegradation of poly-(disulfanyl-n-decane) and of the reference material.

The system allowed a real-time and continuous measurement of the three relevant gases. The results showed that poly-VI-VIM exhibited very low biodegradability under the tested experimental conditions and it did not show any significant difference from the control soil system (data not shown). In contrast, poly-(disulfanyl-n-decane) showed significant biodegradability in the soil (Figures 4–6 and Table 1). A preliminary experiment was done to evaluate the optimal soil biodegradation conditions in terms of pH (Figure 4). In this experiment both poly-(disulfanyl-n-decane) and its monomer were tested for their bioavailability. The biodegradation assessment was done by monitoring the accumulation and evolution rate of CO_2_ and O_2_ respiration of the soil (Figure 4A,B,C,E). The accumulation and evolution rate of CH_4_ was also monitored to see that no release of this potent greenhouse gas (GHG) takes place as part of the biodegradation process (Figure 4C,F). The results indicate a significantly more efficient degradation under basic conditions (pH∼8.5) of the soil than under neutral conditions (pH∼6) (Figure 4). The pH level is known to be an important factor that may have a significant impact on plastic biodegradation in the soil (Emadian et al., 2017; Fernandes et al., 2020; Liu et al., 2021). A recent study found that residues of plastic mulches have a lower negative impact on rice growth under basic conditions (pH 8.5) (Liu et al., 2021). In this study the “inherent” biodegradability was measured rather than a complete “ultimate biodegradability” which is a complete mineralization of the plastic polymer. The “inherent” biodegradability is a sufficient measurement to indicate how much of the plastic polymer is available for a relatively short period of biodegradation.

**TABLE 1:** CO_2_ evolution rate of poly-(disulfanyl-n-decane).

Materials	Average rate (μg/min)	Maximal rate (μg/min)
poly-(disulfanyl-n-decane)	6.88 ± 0.197^b^	39.13 ± 0.895^a^
Crystalline cellulose (References material)	10.50 ± 0.280^a^	25.61 ± 0.458^b^
Blank	4.04 ± 0.152^c^	25.27 ± 3.265^b^

Values are means ± SD (n = 3).

*: The *p* value was obtained by an ANOVA, test with the blank at the same time span. Different letters indicate a significant difference between groups within each time section (α = 0.05).

To get a better understanding of the biodegradation process of poly-(disulfanyl-n-decane) an additional study was performed under optimal pH conditions (pH ∼8.5) and for a longer period (Figure 5). Poly-(disulfanyl-n-decane) showed significantly higher accumulation and evolution rate of CO_2_ and O_2_ respiration than those measured in the control soil respiration (Figure 5).

Both CO_2_ and O_2_ respiration patterns were similar during the three months of the degradation in soil experiment. Around 0.8 and 0.4 g of CO_2_ were produced during the three months from the reference material (cellulose microcrystalline) and the tested poly-(disulfanyl-n-decane) polymer respectively (after subtraction of the results from the soil control). A similar trend was observed for the oxygen consumption with close to 0.6 and 0.2 g of O_2_ consumed during the three months more than the reference material (microcrystalline cellulose) and the tested poly-(disulfanyl-n-decane) polymer respectively (after subtraction of the results from the soil control). It is worth noticing that oxygen consumption is a relative measure of the reduction of oxygen below the atmospheric levels (this is why the results are lower than the CO_2_ production results). The known biodegrading reference material showed as expected the highest biodegradability and reached a 60% degradation in three months (Figure 6), while at the same time the tested poly-(disulfanyl-n-decane) polymer reached only 20% degradation. It is also interesting to note that for an unknown reason the reference material had a certain “lag” phase in the degradation process which was different from the tested material. While at a first glance the observed degradation rate of poly-(disulfanyl-n-decane) seems slow, for many plasticulture applications, including mulching films, it is too fast. Mulching films are used under harsh conditions and their service life ranges from 1 month for short soil treatments to 6 months in some crop protection techniques (Kasirajan and Ngouajio, 2012; Achmon et al., 2018). Along this period of time, these polymer sheets are required to perform with no significant loss of their chemical and physical properties. In order to achieve this, the bio-degradation of the polymer should be by far slower than that of the reference material. The exact optimal degradation conditions test should be done in the specific needed field conditions in the future.

These results are encouraging as the biodegradability of poly-(disulfanyl-n-decane) suggests that it is bioavailable to the soil microbial population in the tested conditions. It was even shown that a significantly maximal rate of respiration was observed with poly-(disulfanyl-n-decane) than with the negative soil control as well as with the positive reference material (Table 1). As this study focuses on the potential of biodegradability rather than on looking at the complete mineralization of the polymer, the test was conducted until 60% degradation of the reference material was reached (Briassoulis and Dejean, 2010). Additionally, all the tested polymers did not show any significant methane production (Figures 4, 5). Methane production from plastic degradation is attracting increasing attention as methane is a potent GHG and it was shown that plastic degradation may have an impact on the overall release of methane into the atmosphere (Royer et al., 2018). The fact that the tested polymer degraded in the soil without any significant production of methane is encouraging in terms of the environmental impact of the biodegradation in the soil of future mulching applications with such polymers.

### Volatiles profile of polymer biodegradation

**FIGURE 7:**
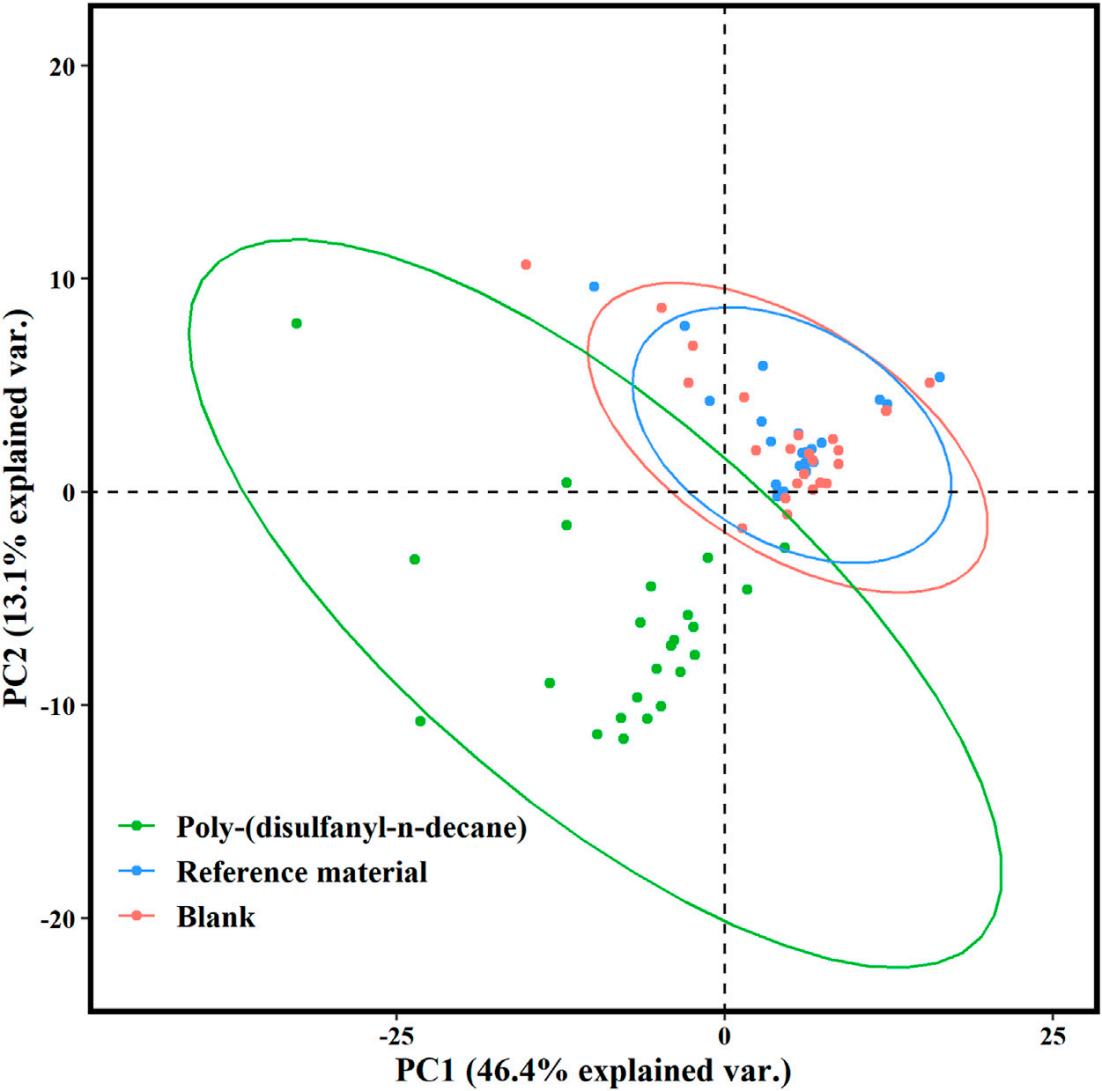
Score plot of the principal component analysis (PCA) for PTR-TOF-MS spectral data of the poly-(disulfanyl-n-decane) polymer, the reference material and the blank after 25 days. The first principal component (PC1) and the second principal component (PC2) described 46.4 and 13.1% of the sample variability, respectively.

**FIGURE 8:**
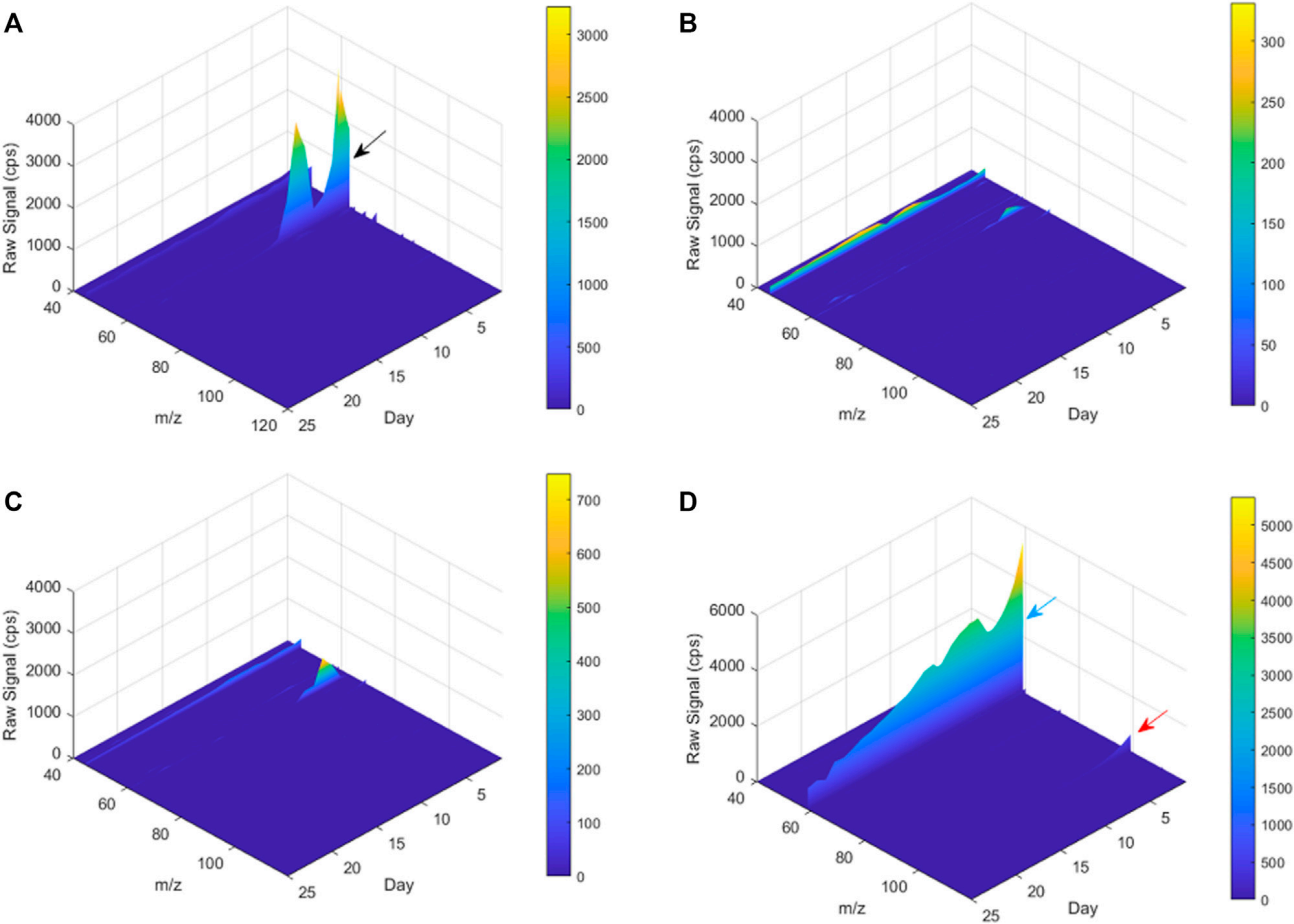
A 3D plot of PTR-TOF-MS spectra for m/z 40–120 from Day 1 to Day 25. **(A)** poly-(disulfanyl-n-decane) and soil mixture, **(B)** reference material and soil mixture, **(C)** only soil (blank), **(D)** only poly-(disulfanyl-n-decane). The black, blue and red arrows mark the peaks of m/z 63, 59 and 99, respectively.

Many studies about biodegradation of plastic polymers in soil have overlooked the characterization of non-CO_2_ volatile products that are being produced during the process. Previous studies recognized the risks of formation of carcinogenic and toxic VOC materialss by plastic degradation (He et al., 2015; Kuchmenko et al., 2020). The present study is among the first to study the profile of VOCs emitted during the biodegradation of plastic polymers in the soil. C^volatile products^ of the poly-(disulfanyl-n-decane) shows a distinct profile of volatiles that is markedly different from the reference material and from the control soil, as can be seen from the PCA plot (Figure 7). Additionally, by using the ability of the PTR-TOF-MS to constantly monitor VOCs emission during the entire biodegradation in soil processes, a plot of the time dependent VOCs emission was created (Figure 8). Interesting phenomena were observed in the production of VOCs over time during the biodegradation in soil process.

The tested poly-(disulfanyl-n-decane) polymer showed a high concentration of a molecule with an *m/z* ratio of 63 (Figure 8A) mainly in the initial first 15 days (this molecule is probably ethanethiol), whereas the reference material showed significantly lower amounts of VOCs production during the tested period and also in the blank soil control (Figures 8B,C). When testing the polymer under basic conditions a high emission with an *m/z* ratio of 59 was observed which might be an indicator for these conditions as it was not observed under natural conditions. The PTR-MS Viewer 3.2 program was used to assess the chemical composition of the VOCs, based on a library of recognized materials (Table 2). Among these recognized molecules, detailed in Table 2, those which could have a special interest are the ones that showed a significantly higher signal coming from the tested poly-(disulfanyl-n-decane) polymer. The major VOCs originating from poly-(disulfanyl-n-decane) polymer were methanethiol, ethanethiol, penta-1,3-diene and 2-pentanone. The presence of these molecules (especially the thiols) might be used as indicators for the biodegradability of poly-(disulfanyl-n-decane) in soil and, as shown in the 3D plot, was predominant at the beginning of the process (Figure 8A). Additional future studies may be able to correlate between the presence and amounts of these molecules and the rate of degradation, as well as to unveil the mechanism of the biodegradation process, in order to see how well the presence and amounts of these molecules can be used to assess the degradation pace of the specific polymer.

**TABLE 2:** Tentative volatile compounds detected during the biodegradation of 1,10-decanedithiol polymer.

*m/z*	Ion formula	Parent formula	Tentative volatile compounds^*^
41^a, b^	(C_3_H_4_)H^+^	C_3_H_4_	Propyne 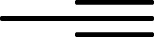
45^a, b^	(CO_2_)H^+^	CO_2_	Carbon dioxide 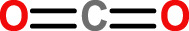
49^a^	(CH_4_S)H^+^	CH_4_S	Methanethiol 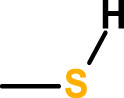
59^b^	(C_3_H_6_O)H^+^	C_3_H_6_O	Acetone 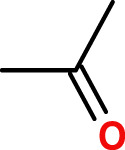
63^a^	(C_2_H_6_S)H^+^	C_2_H_6_S	Ethanethiol 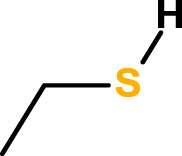
69^a^	(C_5_H_8_)H^+^	C_5_H8	Penta-1,3-diene 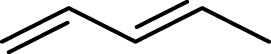
73^a, b^	(C_4_H_8_O)H^+^	C_4_H_8_O	2-Butanone 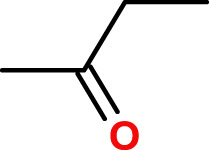
87^a^	(C_5_H_10_O)H^+^	C_5_H_10_O	2-Pentanone 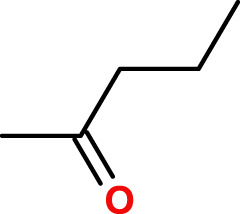

a: The ion intensity was relatively high in the soil mixture with poly-(disulfanyl-n-decane) polymer.

b: The ion intensity was relatively high in the control sample.

*: One tentative volatile compound was suggested according to the chemical formula.

### Culturable microbial profile of polymer biodegradation

**FIGURE 9:**
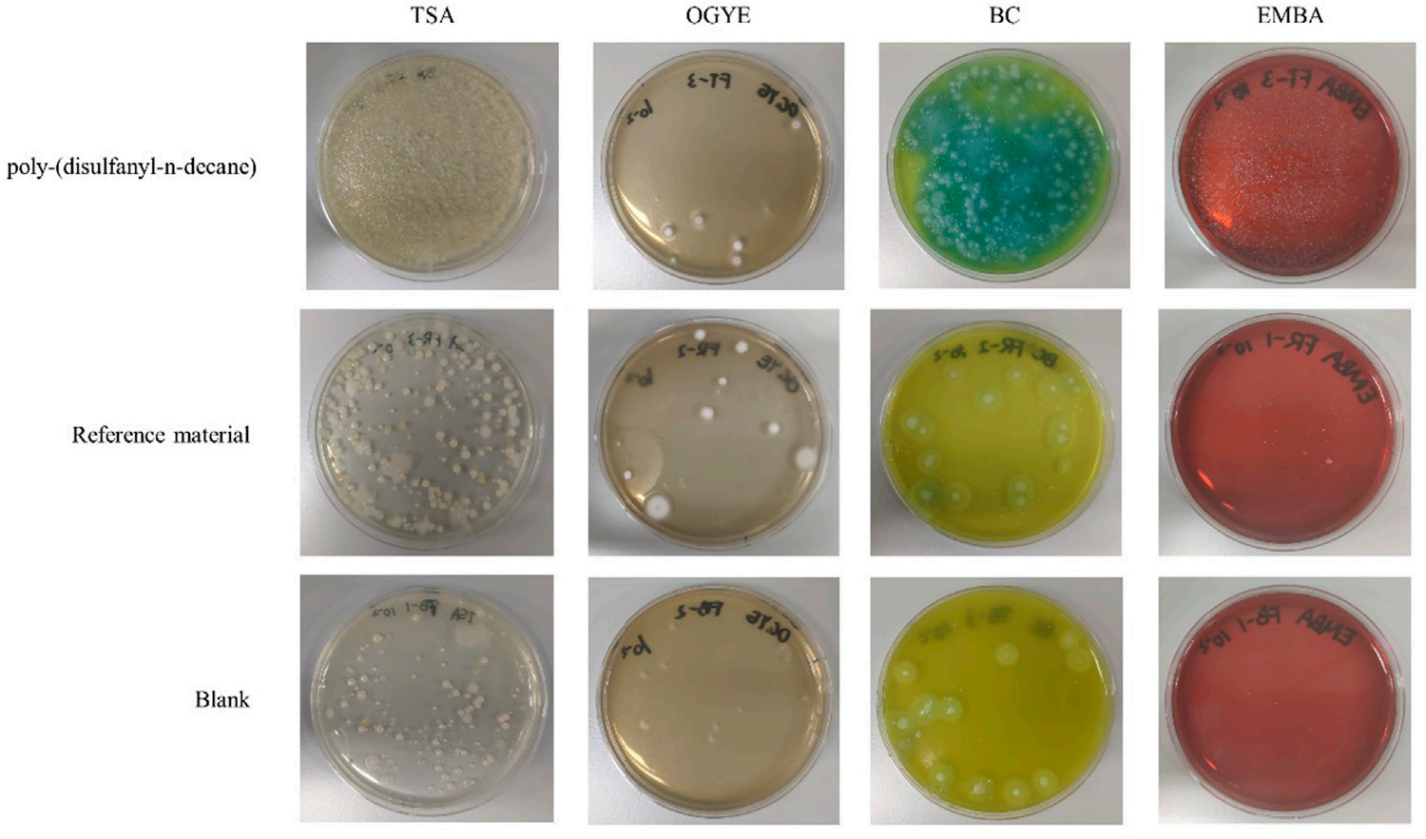
Plating of soil mixtures with a dilution of 10^2^ in Tryptic Soy Agar (TSA), Oxytetracycline Glucose Yeast Extract (OGYE), *Bacillus* cereus Agar (BC) and Eosin Methylene Blue Agar (EMBA) in the end of the biodegradability test.

Even though the soil microbial population responsible for the degradation of the polymer was not the focus of the current study, a general plating test was performed to see if any changes in this population can be observed (Figure 9). The results clearly indicate that the tested polymer had an impact on the soil microbial population expressed by higher numbers observed in the general count of microbes in the TSA plats. While no major differences were observed in the fungal population in the OGYE media, a distinctive difference was observed in both the *Bacillus cereus* agar and the gram-negative Eosin Methylene Blue Agar (EMBA) between the soil with the tested polymer and the positive and negative control soils (Figure 9). It should be noted that these are merely initial results that may suggest that a certain shift in the soil microbial population did occur and that a further study, including next generation DNA sequencing, should be carried out in order to better elucidate these changes.

## Conclusions

The current study looked at two new plastic synthetic polymers which are prototype candidates for future biodegradable plastic mulching films. The initial results showed that one of the tested polymers, poly-(disulfanyl-n-decane), may be bio-available and degrade in the soil under standard conditions, especially under slightly basic soil conditions. Poly-(disulfanyl-n-decane) degrades at a rate of losing ∼20% %wt. of the total polymer mass in three months, which might be too fast for mulching films that must retain their properties for periods of up to 6 months. It is important to note that the tests were done in laboratory conditions and the materials were introduced into the soil which means that a longer period in field conditions should also be tested in the future. Poly-(disulfanyl-n-decane) is essentially a polyethylene into which disulfide bridges were inserted in order to allow biological entities, such as microbes, to break the undigestable long alkane chain into segments that are bioavailable and, as the present work shows, also biodegradable. As the results indicate that poly-(disulfanyl-n-decane) has high bioavailability, the next step will be to further dilute the disulfide bridges in the polyethylene skeleton, thereby creating polymers with mechanical and physical properties that are closer to those of ordinary polyethylene, while achieving at the same time also the desired biodegradability rate for mulching films.

A unique simulation system of biodegradation in field conditions allowed monitoring of the VOCs emitted by the degradation of the poly-(disulfanyl-n-decane) polymer. The system showed a specific volatile “fingerprint” containing methanethiol and ethanethiol which suggest degradation of the polymer through a combination of reduction of the disulfide bridges and cleavage of carbon-carbon bonds in positions α_carbon_-β_carbon_ and β_carbon_-χ_carbon_ relative to the sulfur atom. Monitoring of these materials might serve as markers for determining the rate of biodegradation of this polymer in the future. The microbial population of the soil appears to be impacted by the incorporation of the poly-(disulfanyl-n-decane) polymer into the soil. Future tests will be needed to further tailor this polymer for agricultural usage and to further understand its impact on the soil biological, chemical and physical properties as well as for looking at the fate of the degradation products in real agricultural systems.

## Data Availability

The raw data supporting the conclusions of this article will be made available by the authors, without undue reservation.

## References

[B1] Achmon Y. Dowdy F. R. Simmons C. W. Zohar-Perez C. Rabinovitz Z. Nussinovitch A. (2019). Degradation and bioavailability of dried alginate hydrocolloid capsules in simulated soil system. J. Appl. Polym. Sci. 136, 48142. 10.1002/app.48142

[B2] Achmon Y. Fernández-Bayo J. D. Hernandez K. McCurry D. G. Harrold D. R. Su J. (2017). Weed seed inactivation in soil mesocosms via biosolarization with mature compost and tomato processing waste amendments. Pest Manag. Sci. 73, 862–873. 10.1002/ps.4354 27391139

[B3] Achmon Y. Sade N. Wilhelmi M. del M. R. Fernández-Bayo J. D. Harrold D. R. (2018). Effects of short-term biosolarization using mature compost and industrial tomato waste amendments on the generation and persistence of biocidal soil conditions and subsequent tomato growth. J. Agric. Food Chem. 66, 5451–5461. 10.1021/acs.jafc.8b00424 29763301

[B4] Ahmed T. Shahid M. Azeem F. Rasul I. Shah A. A. Noman M. (2018). Biodegradation of plastics: Current scenario and future prospects for environmental safety. Environ. Sci. Pollut. Res. 25, 7287–7298. 10.1007/s11356-018-1234-9 29332271

[B5] Briassoulis D. Babou E. Hiskakis M. Kyrikou I. (2015). Analysis of long-term degradation behaviour of polyethylene mulching films with pro-oxidants under real cultivation and soil burial conditions. Environ. Sci. Pollut. Res. 22, 2584–2598. 10.1007/s11356-014-3464-9 25192668

[B6] Briassoulis D. Dejean C. (2010). Critical review of norms and standards for biodegradable agricultural plastics part Ι. Biodegradation in soil. J. Polym. Environ. 18, 384–400. 10.1007/s10924-010-0168-1

[B7] Briassoulis D. Mistriotis A. Mortier N. Tosin M. (2020). A horizontal test method for biodegradation in soil of bio-based and conventional plastics and lubricants. J. Clean. Prod. 242, 118392. 10.1016/j.jclepro.2019.118392

[B8] Emadian S. M. Onay T. T. Demirel B. (2017). Biodegradation of bioplastics in natural environments. Waste Manag. 59, 526–536. 10.1016/j.wasman.2016.10.006 27742230

[B9] EN17033 Plastics (2018). EN17033 Plastics - biodegradable mulch films for use in agriculture and horticulture - requirements and test methods. Available at: https://www.en-standard.eu/din-en-17033-plastics-biodegradable-mulch-films-for-use-in-agriculture-and-horticulture-requirements-and-test-methods.

[B10] Fernandes M. Salvador A. Alves M. M. Vicente A. A. (2020). Factors affecting polyhydroxyalkanoates biodegradation in soil. Polym. Degrad. Stab. 182, 109408. 10.1016/j.polymdegradstab.2020.109408

[B11] He Z. Li G. Chen J. Huang Y. An T. Zhang C. (2015). Pollution characteristics and health risk assessment of volatile organic compounds emitted from different plastic solid waste recycling workshops. Environ. Int. 77, 85–94. 10.1016/j.envint.2015.01.004 25667057

[B12] Huang Y. Liu Q. Jia W. Yan C. Wang J. (2020). Agricultural plastic mulching as a source of microplastics in the terrestrial environment. Environ. Pollut. 260, 114096. 10.1016/j.envpol.2020.114096 32041035

[B13] ISO (2019). Plastics — determination of the ultimate aerobic biodegradability of plastic materials in soil by measuring the oxygen demand in a respirometer or the amount of carbon dioxide evolved. Available at: https://www.iso.org/standard/74993.html. 26

[B14] Kasirajan S. Ngouajio M. (2012). Polyethylene and biodegradable mulches for agricultural applications: A review. Agron. Sustain. Dev. 32, 501–529. 10.1007/s13593-011-0068-3

[B15] Kuchmenko T. Umarkhanov R. Lvova L. (2020). E-nose for the monitoring of plastics catalytic degradation through the released Volatile Organic Compounds (VOCs) detection. Sensors Actuators B Chem. 322, 128585. 10.1016/j.snb.2020.128585

[B16] Kyrikou I. Briassoulis D. (2007). Biodegradation of agricultural plastic films: A critical review. J. Polym. Environ. 15, 125–150. 10.1007/s10924-007-0053-8

[B17] Liu Y. Huang Q. Hu W. Qin J. Zheng Y. Wang J. (2021). Effects of plastic mulch film residues on soil-microbe-plant systems under different soil pH conditions. Chemosphere 267, 128901. 33248737 10.1016/j.chemosphere.2020.128901

[B18] Royer S.-J. Ferrón S. Wilson S. T. Karl D. M. (2018). Production of methane and ethylene from plastic in the environment. PLoS One 13, e0200574. Available at: 10.1371/journal.pone.0200574 30067755 PMC6070199

[B19] Sander M. (2019). Biodegradation of polymeric mulch films in agricultural soils: Concepts, knowledge gaps, and future research directions. Environ. Sci. Technol. 53, 2304–2315. 10.1021/acs.est.8b05208 30698422

[B25] Sevilia S. Parvari G. Bernstein J. Fridman N. Grinstein D. Gottlieb L. (2022). Imidazolium based energetic materials. Chem. Select 7, e202200322. 10.1002/slct.202200322

[B27] Tada Y. Yamamoto T. Tezuka Y. Kawamoto T. Mori T. (2012). Effective synthesis and crystal structure of a 24-membered cyclic decanedisulfide dimer. Chem. Letters 41 (12), 1678–1680. 10.1246/cl.2012.1678

[B20] Thomas Z. M. Arno S. Frederick N. T. Rebekka B. Dagmar W. Michael W. (2021). Biodegradation of synthetic polymers in soils: Tracking carbon into CO2 and microbial biomass. Sci. Adv. 4, eaas9024. 10.1126/sciadv.aas9024 PMC6059733 30050987

[B26] Zertal Y. Yong M. Levi A. Sevilia S. Tsoglin A. Parvari G. (2022). Alkyl vinyl imidazolium ionic liquids as fuel-binders for photo-curable energetic propellants. Adv. Funct. Mat.. In press. 10.1021/acsapm.2c00499

[B21] Zhang H. Miles C. Ghimire S. Benedict C. Zasada I. DeVetter L. (2019). Polyethylene and biodegradable plastic mulches improve growth, yield, and weed management in floricane red raspberry. Sci. Hortic. 250, 371–379. 10.1016/j.scienta.2019.02.067

[B22] Zhou B. Wang J. Zhang H. Shi H. Fei Y. Huang S. (2020). Microplastics in agricultural soils on the coastal plain of Hangzhou Bay, east China: Multiple sources other than plastic mulching film. J. Hazard. Mat. 388, 121814. 10.1016/j.jhazmat.2019.121814 31843412

[B23] Zumstein M. T. Narayan R. Kohler H.-P. E. McNeill K. Sander M. (2019). Dos and do nots when assessing the biodegradation of plastics. 10.1021/acs.est.9b04513 31418543

[B24] Zurier H. S. Goddard J. M. (2021). Biodegradation of microplastics in food and agriculture. Curr. Opin. Food Sci. 37, 37–44. 10.1016/j.cofs.2020.09.001

